# Electromagnetic analysis of the lasing thresholds of hybrid plasmon modes of a silver tube nanolaser with active core and active shell

**DOI:** 10.3762/bjnano.10.28

**Published:** 2019-01-28

**Authors:** Denys M Natarov, Trevor M Benson, Alexander I Nosich

**Affiliations:** 1 Laboratory of Micro and Nano Optics, Institute of Radio-Physics and Electronics NASU, vul. Proskury 12, Kharkiv 61085, Ukraine; 2 George Green Institute for Electromagnetics Research, The University of Nottingham, University Park, Nottingham, NG7 2RD, UK

**Keywords:** hybrid localized plasmon mode, nanolaser, nanotube, threshold

## Abstract

Results from the electromagnetic modeling of the threshold conditions of hybrid plasmon modes of a laser based on a silver nanotube with an active core and covered with an active shell are presented. We study the modes of such a nanolaser that have their emission wavelengths in the visible-light range. Our analysis uses the mathematically grounded approach called the lasing eigenvalue problem (LEP) for the set of the Maxwell equations and the boundary and radiation conditions. As we study the modes exactly at the threshold, there is no need to invoke nonlinear and quantum models of lasing. Instead, we consider a laser as an open plasmonic resonator equipped with an active region. This allows us to assume that at threshold the natural-mode frequency is real-valued, according to the situation where the losses, in the metal and for the radiation, are exactly balanced with the gain in the active region. Then the emission wavelength and the associated threshold gain can be viewed as parts of two-component eigenvalues, each corresponding to a certain mode. In the configuration considered, potentially there are three types of modes that can lase: the hybrid localized surface plasmon (HLSP) modes of the metal tube, the core modes, and the shell modes. The latter two types can be kept off the visible range in thin enough configurations. Keeping this in mind, we focus on the HLSP modes and study how their threshold gain values change with variations in the geometrical parameters of the nanotube, the core, and the shell. It is found that essentially a single-mode laser can be designed on the difference-type HLSP mode of the azimuth order *m* = 1, shining in the orange or red spectral region. Furthermore, the threshold values of gain for similar HLSP modes of order *m* = 2 and 3 can be several times lower, with emission in the violet or blue parts of the spectrum.

## Introduction

The promise of greatly enhanced light–matter interaction in nanostructured metal configurations, combined with controlled precision of their fabrication, has already turned plasmonics into a very dynamic research area within contemporary optics and photonics. The physical basis of this enhanced interaction is provided by the existence of the very slow (in the phase-velocity sense) surface-plasmon waves guided by metal–dielectric interfaces or thin metal layers, and their standing-wave counterparts, localized surface plasmon (LSP) modes on metal particles and wires of deeply sub-wavelength (sub-λ) size. This phenomenon occurs due to the specific properties of the complex dielectric functions of metals in the optical range, namely their negative real-part values, Re ε_met_(λ) < 0 [1–2]. The resonances on the LSP modes are already used in the design of nanoantennas and nanosensors in which small changes in the refractive index of the host medium allow for a direct measurement of low concentrations of various substances. More recently, a new theme of research effort has appeared around the LSP modes: the analysis and design of plasmonic nanolasers (also called spasers) where a nanoscale metal particle, wire, strip or shell serves as a miniature open resonator, and the presence of the active region can be provided in a variety of ways. After initial theorizing in the mid-2000s, this has led to the experimental demonstration of the smallest plasmonic laser in a random solution of colloidal gold nanospheres enveloped with dye-doped silica shells [3]. Today, plasmonic nanolasers attract great attention in research. A number of publications have dealt with the modeling of nanolasers based on metal nanoparticles and nanowires equipped with active cores or shells [4–9]. Note that in [4], the authors studied the effect of a metal tube on the lowest-order modes of the active core while the presence of the plasmon modes of the tube itself was neglected. More recently, the attention of researchers became focused on lasing in periodic arrays of metal nanoparticles supported by or immersed into an active layer [10–13] or with other active region configurations [14].

In this paper, we explore the modes of a silver nanotube as a promising nanocavity, which is able to support transverse HLSP modes at wavelengths strongly dependent on the tube thickness. This is in sharp contrast to the LSP modes of a solid circular metal wire that are all close, in wavelength, to the roots of the textbook equation Re ε_met_(λ) = −ε_host_ where ε_host_ > 0 is the relative dielectric permittivity of the host medium – see [1–2] for details. If the host medium is air, then the corresponding wavelength is found in the ultraviolet range for silver and in the green range for gold where the bulk losses in metals are considerable. This means that, to achieve lasing, any natural mode of a plasmonic laser has to overcome the losses in the metal element (plus much smaller radiation losses) that places the threshold values of material gain at the same level as Im ε_met_. As explained in [15–16], by manipulating the nanotube thickness, one can shift some of the low-order hybrid modes to the orange and even the red parts of the visible spectrum. Here, the bulk losses in metals are much smaller than in the ultraviolet part; this should result in lower thresholds for these modes. But it appears that these thresholds have not been accurately quantified so far.

Our instrument for the analysis of the threshold conditions is the LEP formalism, which is described in detail in [17] for arbitrary laser models. This is the eigenvalue (source-free) electromagnetic field boundary problem specifically tailored to provide both the modal wavelengths and the associated values of threshold material gain in the active region. This is because, in contrast to the conventional eigenvalue problem aimed at the complex modal frequencies (and associated Q-factors) for a passive optical cavity, LEP fully takes into account the size, shape and location of the active region. As shown in [17], every LEP eigenvalue automatically satisfies the “gain = loss” condition. Therefore the LEP formalism is, in fact, the full-wave classical (i.e., purely electromagnetic) laser threshold theory equally valid for any two-dimensional (2D) and three-dimensional (3D) configuration. Here it should be noted that the so-called “semi-classical theory,” developed at the onset of laser studies when early laser resonators were measured in thousands of λ, coincides with the LEP for the simplified one-dimensional laser models, which involve only flat-layered infinite-width microcavities [18].

To date, the LEP approach has been successfully applied to a variety of 2D microlasers, which appear as reasonable approximations of 3D configurations shaped as thin flat “disks” or “patches”: single fully active microcavities in the form of a circle [19], kite [20], and square [21], active cyclic photonic molecules [22–23], active circular disks with passive annular Bragg reflectors [24], and partially active circular and elliptic cavities [25–26]. More recently the LEP was applied to the modes of a single plasmonic nanowire [5] and a single plasmonic nanostrip [8] placed into an active circular shell. Alternatively, such configurations can be seen as a quantum wire loaded with a plasmonic open resonator. An infinite array of circular quantum nanowires was considered with LEP in [27] where it was shown that such a periodic open active resonator can support so-called lattice modes with ultra-low thresholds and wavelengths located near to the Rayleigh anomalies. A similar LEP-based study of the lasing modes of an infinite binary grating of circular silver and quantum nanowires was published in [14] where the thresholds of the LSP modes were found to be higher than those of the lattice modes.

Note also that there exist other LEP-like formulations aimed at the extraction of mode threshold [28–32]; some of them differ from LEP only by the choice of the material-gain parameter, which can be the imaginary part of the dielectric permittivity (because Im ε_a_ = 2αγ, where α is assumed known) or the product of the wavenumber and the imaginary part of the refractive index (*g* = *k*γ, *k* = 2π/λ) – this choice is typical for semi-classical laser theory. In any case the principal step is assuming the threshold value of gain to be unknown and finding it as an eigenvalue. This is fully adequate to the fundamental observation, known from the onset of laser research, that the thresholds of lasing are specific to each mode and hence are closely tied to the mode field pattern and its overlap with the active region.

Thus, the goal of this work is to analyze the threshold conditions of the HLSP modes of a silver nanotube laser with double active region. To the best of our knowledge, although these modes have been known in the passive applications such as refractive-index sensors, they have not been studied yet from the viewpoint of lasing.

## Results and Discussion

### Lasing eigenvalue problem

**Figure 1:**
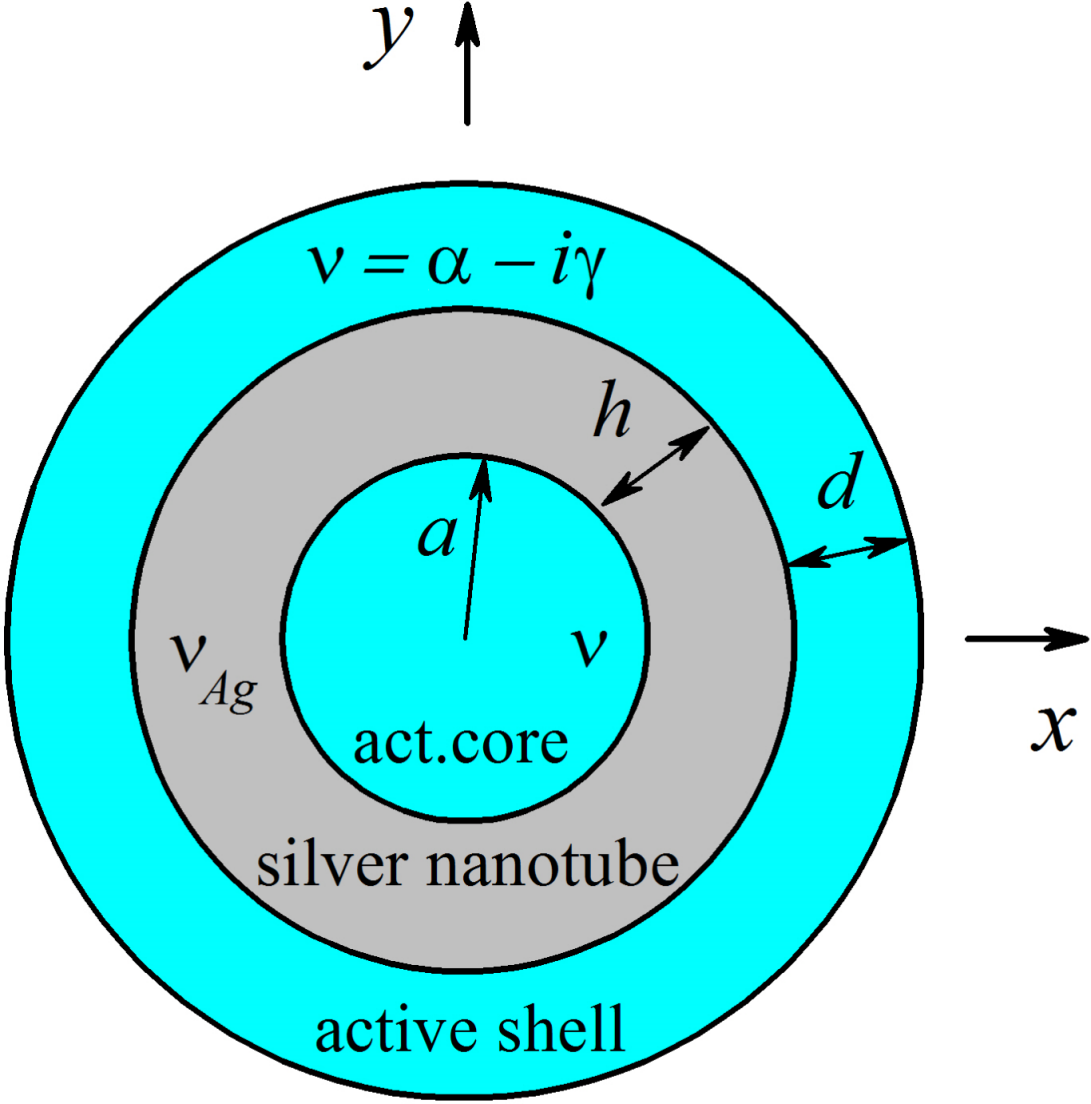
Cross section of a nanolaser built on a silver nanotube with an active core and an active shell.

Figure 1 presents a cross-sectional view of the nanoscale laser based on a silver nanotube. It is assumed that the tube is infinite along the *z*-axis, and that the electromagnetic field does not depend on *z*; hence the problem under consideration is a 2D problem. To make a resonator able to emit electromagnetic waves non-attenuating in time, one must equip it with an active zone filled with a material possessing optical gain. Such material can be a semiconductor, a dye-doped polymer, or a material doped with ions of erbium or some other rare-earth elements. All of them are able, under pumping, to demonstrate the inverse population of electronic levels and the stimulated emission of light. In the model of a nanolaser considered here, it is assumed that the active zone has the form of concentric circular shell of thickness *d* and that the same active material also fills the inner core of the tube that has the radius *a*.

This configuration of the active region is selected as the most favorable for achieving lower thresholds of the LSP modes. Such anticipation is based on the finding of [17] (see Equation 36 there): low threshold needs good overlap of the active region with the electric field of mode, and on the known property of LSP modes to “stick” to the metal–dielectric boundary. Indeed, as the hybrid LSP modes of a nanothin tube have their maxima at both boundaries, the gain in only one region (core or shell) will entail roughly twice higher thresholds than the gain in both regions (see also Equation 1 of the present paper and its derivation).

Denote by *U* the component of the magnetic field *H*_z_. Assuming that the field is time-harmonic and depends on time as *e*^−iω^*^t^*, the LEP implies that the function *U* must satisfy the 2D Helmholtz equation with the corresponding refractive indices in each of the regions, i.e., complex ν = α − iγ in the core and the shell active regions, where α is the known refractive index and γ is the unknown material gain, the known complex ν_met_(λ) of the tube metal, and 1 in the outer air region. On the boundaries of the partial regions, the conditions of continuity of two tangential field components, *H*_z_ and *E*_φ_ have to be satisfied. In addition, the Sommerfeld radiation condition at infinity and the condition of local power finiteness are imposed. We assume that the material gain is uniformly distributed within the active regions and is independent of the wavelength. For a mode on the threshold of lasing, the wavelength is assumed to be real-valued. The task is to find two numbers, the mode wavelength and the associated value of material gain, which are the components of the LEP eigenpairs (λ*_N_*, γ*_N_*), where *N* is a generic mode number.

For generality, consider a general configuration, which has *S* concentric circular boundaries and, respectively, *S* + 1 regions, where *s* = 1 corresponds to the central region, and *s* = *S* + 1 to the outer region. Introducing the polar coordinates (*r*, φ) and denoting the radius of the *s*-th boundary and the refractive index in the *s*-th region as *a*_s_ and ν_s_, respectively, we can write the field in each of the regions as a Fourier series in terms of the azimuth functions,

[2] $$\begin{array}{c} U^{s}\left(r,\text{φ}\right)=\left[A_{0}^{s}J_{0}\left(k{\text{ν}}_{s}r\right)+B_{0}^{s}H_{0}\left(k{\text{ν}}_{s}r\right)\right]\\ +\sum_{m=1}^{\infty }\left[A_{m}^{s}J_{m}\left(k{\text{ν}}_{s}r\right)+B_{m}^{s}H_{m}\left(k{\text{ν}}_{s}r\right)\right]\left\{\begin{array}{c} \cos m\varphi \\ \sin m\varphi \end{array}\right\}, \end{array}$$

where *J_m_* and *H_m_* are the Bessel and the first-kind Hankel functions, respectively, and 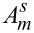 and 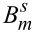 are unknown coefficients. From the condition of local power finiteness and the radiation condition at infinity, it follows that the field in the central region must contain only the Bessel functions, and in the outer space only the Hankel functions, that is 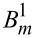 = 0 and 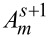 = 0. In view of the orthogonality of the azimuth functions with different indices *m* in Equation 2, the corresponding mode families can be analyzed separately. Note that all modes with *m* > 0 are twice degenerate.

To find the coefficients 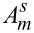 and 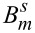, we use the boundary conditions, which generate a pair of equations at each boundary, *r* = *a_s_*, where *s* = 1,2,…*S*, of the following form:

[3] $$\begin{array}{l} A_{m}^{s}J_{m}\left(k{\text{ν}}_{s}a_{s}\right)+B_{m}^{s}H_{m}\left(k{\text{ν}}_{s}a_{s}\right)A_{m}^{s+1}\\ -J_{m}\left(k{\text{ν}}_{s+1}a_{s}\right)-B_{m}^{s+1}H_{m}\left(k{\text{ν}}_{s+1}a_{s}\right)=0, \end{array}$$

[4] $$\begin{array}{l} \frac{1}{{\text{ν}}_{s}}\left[A_{m}^{s}{J^{\prime }}_{m}\left(k{\text{ν}}_{s}a_{s}\right)+B_{m}^{s}{H^{\prime }}_{m}\left(k{\text{ν}}_{s}a_{s}\right)\right]\\ -\frac{1}{{\text{ν}}_{s+1}}\left[A_{m}^{s+1}{J^{\prime }}_{m}\left(k{\text{ν}}_{s+1}a_{s}\right)+B_{m}^{s+1}{H^{\prime }}_{m}\left(k{\text{ν}}_{s+1}a_{s}\right)\right]=0\;. \end{array}$$

Collecting all such equations together, and denoting *x*_2_*_s_*_−1_ = 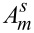 and *x*_2_*_s_* = 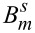 for *s* = 1,2,…*S*, we obtain, for each fixed *m*, a matrix equation of the order 2*S*,

[5] $${\left\{F_{pq}(m;\lambda ,\text{γ})\right\}}_{p,q=1}^{2S}X=0,\;\;\;\;m=0,1,2,...\;,$$

where *F_pq_*(*m*; λ, γ) is the matrix operator built from Equation 3 and Equation 4 written for all *S* boundaries and 
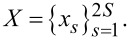
 The elements *F_pq_* can be easily computed with accuracy to machine precision. The LEP eigenvalues sought are the roots of the corresponding determinant equations,

[6] $$\det{\left\{F_{pq}(m;\lambda ,\gamma )\right\}}_{p,q=1}^{2S}=0,\;\;\;\;m=0,1,2,...\;.$$

The configuration depicted in Figure 1 has *S* = 3 boundaries, and the corresponding geometrical and material parameters are *a*_1_ = *a*, *a*_2_ = *a* + *d*, *a*_3_ = *a* + *d* + *h*, ν_1_ = ν_3_ = α – iγ, and ν_2_ = ν_met_.

**Figure 2:**
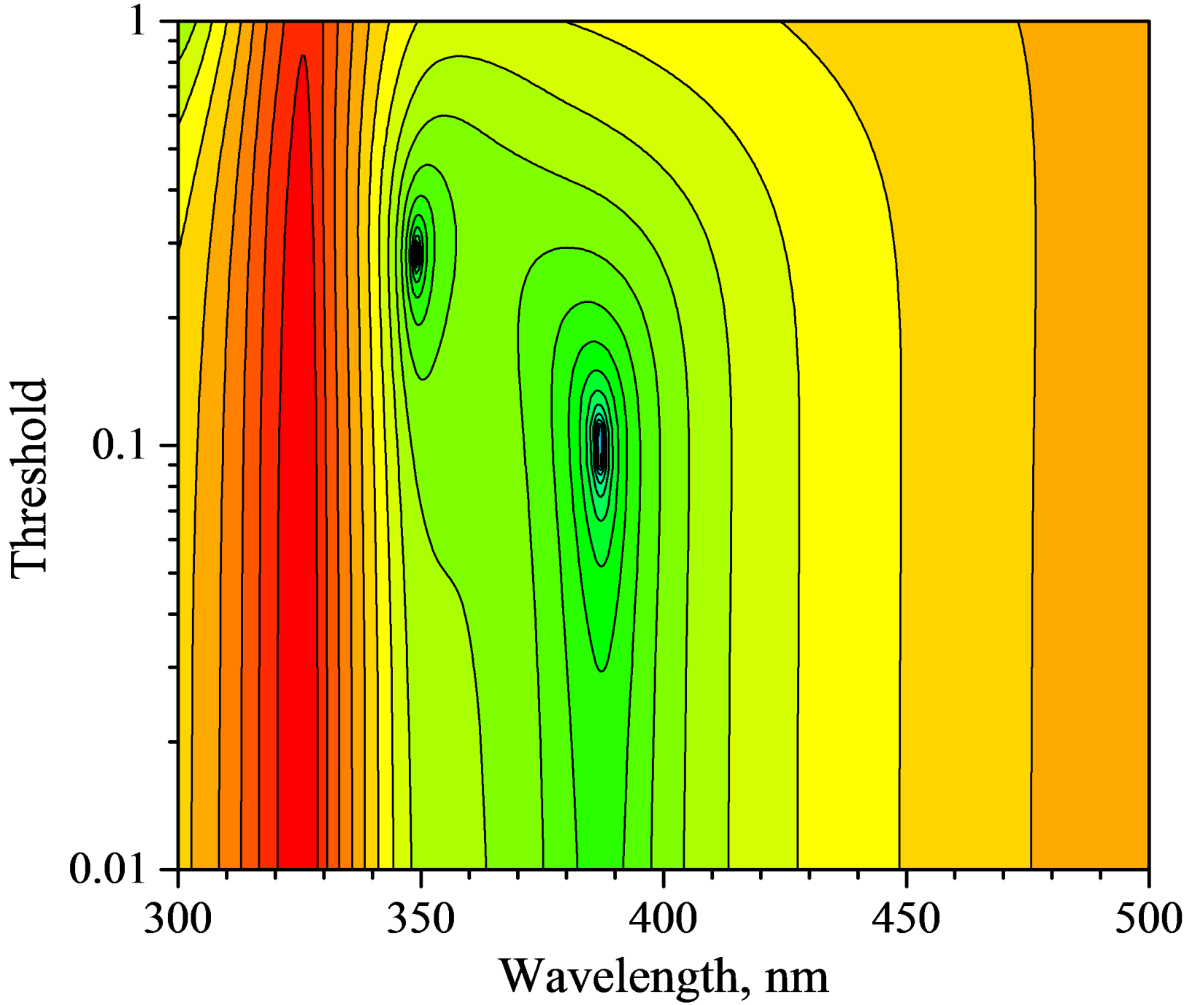
Relief of the function *D*_2_(λ, γ) for a nanotube with *a* = *h* = 30 nm, *d* = 10 nm, α = 1.5.

To solve the transcendental Equation 6 numerically, we use an iterative Newton-type algorithm that needs some initial guess values of the unknown wavelength λ and threshold gain γ. Because of the strong dispersion of the dielectric permittivity of silver, it is convenient to take these initial values after plotting the color map (relief) of the function *D_m_* = |det[*F_sp_*(*m*; λ, γ)]|. An example of such a map for the function *D*_2_(λ, γ) is presented in Figure 2. As one can see, there are two clearly visible “holes,” where this function is close to zero. One of them occurs at a wavelength near to 350 nm and the other at a wavelength around 385 nm. They indicate two possible LEP eigenvalues corresponding to the quadrupole-type HLSP modes 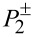, as shall be discussed further.

In the computations, an important consideration is the data for the complex dielectric permittivity of silver. Note that, according to [5], a simple Drude formula does not accurately approximate the experimental data of [33], especially in the violet part of visible spectrum, where at least some of the HLSP modes of the silver tube have their emission wavelengths. The modifications of the Drude formula presented in [34] are more accurate, but yield a non-physical negative Im ε_met_ in silver at longer wavelengths. Therefore we use the measured values of dielectric function of silver from [33] and interpolate them with the aid of Akima splines, to determine the complex permittivity at any wavelength between the measured values.

Note also that, according to the research reported, e.g., in [35], non-local effects in metallic particles have to be taken into account only if their dimensions become smaller than 3–5 nm. Otherwise one can characterize the complex dielectric permittivity using its bulk value.

### Complex Poynting theorem for the modes of a nanolaser

The complex Poynting theorem is the direct consequence of the Green’s formula applied to the functions, which solve the Maxwell equations. In plane-wave scattering, the optical theorem is obtained as the real part of the complex Poynting theorem when it is applied to the total field, i.e., to the sum of the incident and scattered field, and its complex conjugate [1].

In the LEP, there is no incident field however the same complex Poynting theorem can be used as well. As shown in [17], if applied to a lasing mode field *U* and its complex conjugate counterpart U*, its real part, i.e., the optical theorem, leads to the well-known “gain = loss” condition, widely used in the so-called “semi-classical laser theory”. Thus, this condition is a direct consequence of the fact that *U* is a solution of the LEP and so it is valid for an arbitrary laser configuration, i.e., has universal validity, not restricted to “semi-classical theory”. If, as in the case of a plasmonic laser considered here, a lossy region *V*_abs_ is present in addition to the active region *V*_gain_, then the Optical Theorem takes the following most general form:

[7] $$W_{N}^{\text{(gain)}}=W_{N}^{\text{(abs)}}+W_{N}^{\text{(rad)}},$$

where 
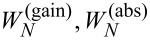
 and 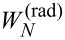 are the powers generated in, absorbed in and radiated away from the laser cavity. In line with the classical electromagnetics of time-harmonic fields (depending on time as *e*^−iω^*^t^*), these quantities are

[8] WN(gain)=−(Z0/2)Imεa∫Vgain|EN|2dv,

[9] WN(abs)=(Z0/2)Imεmet(λ)∫Vabs|EN|2dv,

[10] WN(rad)=ReΠN/kN,    ΠN=(1/2)∫S(EN×HN*)ds,

where *Z*_0_ is the free-space impedance, ε = ν^2^, *S* is a closed surface, which contains all resonator elements, and 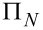 is the mode complex Poynting vector.

If the active region does not fill the whole open resonator (this is the case for the configuration shown in Figure 1), then it is convenient to introduce the quality factors linked to the absorption in metal and the radiation into the host medium, respectively,

[11] $$Q_{N}^{\text{(abs)}}=W_{N}/W_{N}^{\text{(abs)}},\;Q_{N}^{\text{(rad)}}=W_{N}/W_{N}^{\text{(rad)}},$$

where the power stored in the open resonator is expressed as

[12] WN=(Z0/2)Re[d(ε(ω)ω)/dω]∫Vmin|EN|2dv,

and *V*_min_ is the volume of open resonator, that is the inner domain of the minimum circle containing all of the resonator elements [17]. If the mode electric field is normalized by its maximum magnitude value, then *W_N_* coincides with the effective mode volume – this quantity is an important “effective” footprint of both the cavity geometry and composition and the given lasing mode field pattern.

In our case, *V*_min_ = *V*_gain_ + *V*_abs_ and 
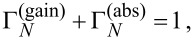
 however, the active region consists of two separate domains (the core and the shell) and hence *V*_gain_ = *V*_core_ + *V*_shell_. Therefore each of the quantities 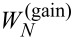 and 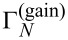 is also a sum of two partial values, so it is convenient to introduce the overlap coefficients between each part of the active region and the mode electric field,

[13] $$\begin{array}{l} \Gamma_{N}^{\text{(gain}\text{,core)}}=W_{N}^{\text{(gain}\text{,core)}}/W_{N},\\ \Gamma_{N}^{\text{(gain}\text{,shell)}}=W_{N}^{\text{(gain}\text{,shell)}}/W_{N}. \end{array}$$

Then the “gain = loss” Equation 7 takes the following form:

[14]
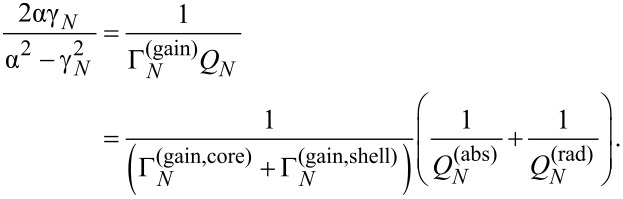


Equation 14 is, of course, simply a re-written optical theorem and hence it is valid for any mode of any laser containing both lossy elements and active regions. Furthermore the quantities 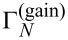 and *Q_N_* (and their components) appearing on the right-hand side of this equation also depend indirectly on γ*_N_*; this hinders its understanding. However, Equation 14 takes an extremely “transparent” and handy form if the mode threshold gain is assumed small, γ*_N_* << 1. Then, after taking into account that both 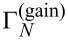 and *Q_N_* are quadratic with respect to the field amplitude, we can replace them, with an error of the order of 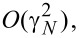 by the same values calculated in the absence of gain (γ*_N_* = 0), 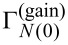 and *Q_N_*_(0)_. Thus,

[1] $$\gamma_{N}=\frac{\left(1/2\right)\alpha }{\Gamma_{N(0)}^{\text{(gain)}}Q_{N(0)}}+O\left(\gamma_{N}^{2}\right).$$

Note that if γ*_N_* << 1, then Equation 14 can also be cast to the form given in Equation 15; this is equivalent to Equation 1 but presented in terms of the dielectric permittivity of the gain material in the active region,

[15]
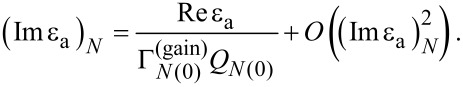


Thus the lowest-threshold mode is not the one with the highest quality factor, *Q_N_*_(0)_, of the pump-off cavity, but the mode with the largest product of 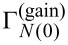 and *Q_N_*_(0)_.

The expression obtained, in either form, communicates the fundamental engineering rule for the design of low-threshold laser: take the highest-Q mode of the cavity and equip it with the active region matching its E-field pattern as closely as possible, to provide 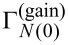 ≈ 1. In the case of the nanotube plasmonic laser studied here, the HLSP modes have the maximum E-field values on two boundaries of the tube, the inner and the outer. This explains our choice of a two-part active region, the core and the shell, because if we leave only one of them, the overlap coefficient will be at best close to 1/2.

It should be noted that in the presence of a lossy metal, 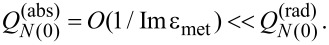 Then, even if a good overlap is achieved, so that 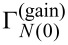 ≈ 1, the threshold gain of any plasmonic mode is of the same order as Im ε_met_(λ). Still it depends on the type of the mode and the tube thickness. More accurate estimation is obtained from Equation 7 using the quasi-static expressions for the HLSP mode fields of the metal nanotube, derived in [36]. The cumbersome algebraic expressions can be simplified if *h*/*a* << 1 that yields the following lower-bound formula for the most important “difference” modes, 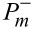:

[16] γ>Imεmet(λ)h4αa.

From Equation 16, one can conclude that taking extremely thin-wall metal tubes is a way to a significant lowering of the threshold. However, this is not true as, if *h* ≤ 5 nm, then the permittivity must be corrected for non-local effects, which leads to considerably larger values of Im ε_met_ [35].

### Numerical analysis of hybrid plasmon mode thresholds

In this section we present the study of the emission wavelengths and the threshold values of gain, together with the fields of the lasing modes of a nanolaser based on a silver nanotube with a thin active shell and an active core. All computations have been performed using Equation 6 as explained in sub-section lasing eigenvalue problem. We assume that the gain material is nonmagnetic and has a refractive index α = 1.5 (hence the relative dielectric permittivity is ε = 2.25) that does not depend on the wavelength.

It is a well-established fact that on thin metal nanotubes, the thickness of which is comparable to the skin-depth thickness in the optical range (about 10 to 20 nm), the modes of the outer and inner boundaries hybridize [15]. This means that they form pairs, 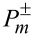, in which the H-field of one mode is the sum and the other is the difference of the fields of the two modes of each boundary in the absence of the other. Thus, every HLSP mode can be viewed as a supermode, with H-field maxima at both boundaries and either zero or non-zero values of the field in the middle of thin-wall silver tube (i.e., between *r* = *a* and *r* = *a* + *h*). Their emission wavelengths are found from a quasi-static equation [16]. For instance, if the outer medium is uniform and its material is the same as that of the core, then such equation for every azimuth order *m* = 1,2,… is

[17] Reεmet(λPm±)≈−εhost±2εhost[(1+ha)m±1]−1.

Note that if the tube wall becomes thicker, *h* >> *a*, then two HLSP sister-mode wavelengths come together to the same quasi-static value known for both the solid metal wire and the void in the metal medium, Re ε_met_(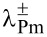) = −ε_host_. Note that the configuration of Figure 1 has a finite-thickness shell covering the metal tube from the outside, and therefore corrections to Equation 17 of the order of *O*[*d^m^*/(*a* + *h* + *d*)*^m^*] can be anticipated.

However, as it was shown in [5], if the shell thickness, *d*, becomes close to the half-wavelength in the shell material, then the active shell can support its own lasing modes in the optical wavelength range (shell modes). The same is true for the modes of the void, i.e., of the active core, studied in [4] (HLSP modes were not studied in [4]). Therefore, to exclude the appearance of shell and core modes in this range, in the computations we will consider only nanotube lasers with sufficiently thin outer active shells of the thickness *d* from 10 nm to 30 nm, and the inner active cores of the radius *a* from 10 nm to 50 nm. Note that the threshold values of gain, γ, of the shell modes are typically higher than those of the LSP modes, apparently because of more significant radiation losses [5].

In addition to the shell and core modes, the existence of the other “secondary” plasmon modes of the real-material (i.e., not Drude) silver wire with an active shell was shown in [5]. They have emission wavelengths in the deep ultraviolet; they are not studied here as they have very large emission thresholds, well above γ = 1.

**Figure 3:**
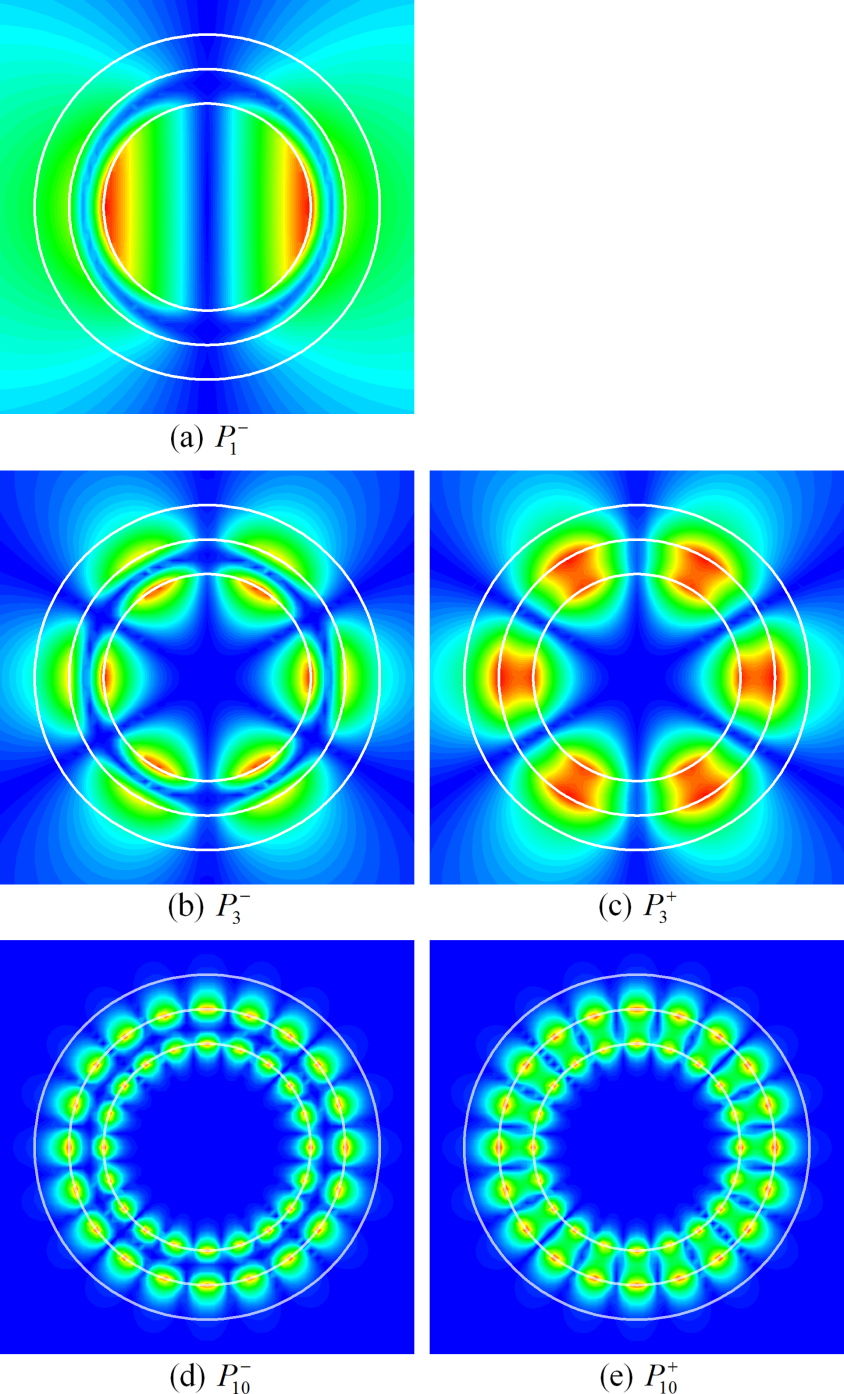
Near H-fields of the eigenmodes of thin-wall nanotube, *a* = 30 nm and *h* = *d* = 10 nm: (a) 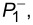 λ = 565.031 nm, γ = 0.122, (b) 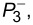 λ = 414.238 nm, γ = 0.035, (c) 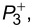 λ = 335.46 nm, γ = 0.269, (d) 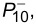 λ = 363.737 nm, γ = 0.073, (e) 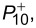 λ = 354.361 nm, γ = 0.105.

Figure 3 presents the |*H_z_*| near-field portraits of several HLSP lasing modes, 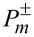, with azimuthal indices *m* = 1, 3 and 10, of a thin-wall (*h* = 10 nm) silver nanotube laser; these modes are also marked on the trajectories to be presented in Figures 5 to 7.

The features of the mode fields that explain our notations are clearly visible in these portraits: this is the presence of either zero fields (dark rings) or non-zero fields (2*m* bright spots) in the middle of the nanotube wall (see also [36]). The field maxima stick to both the outer and the inner boundary of the nanotube.

**Figure 4:**
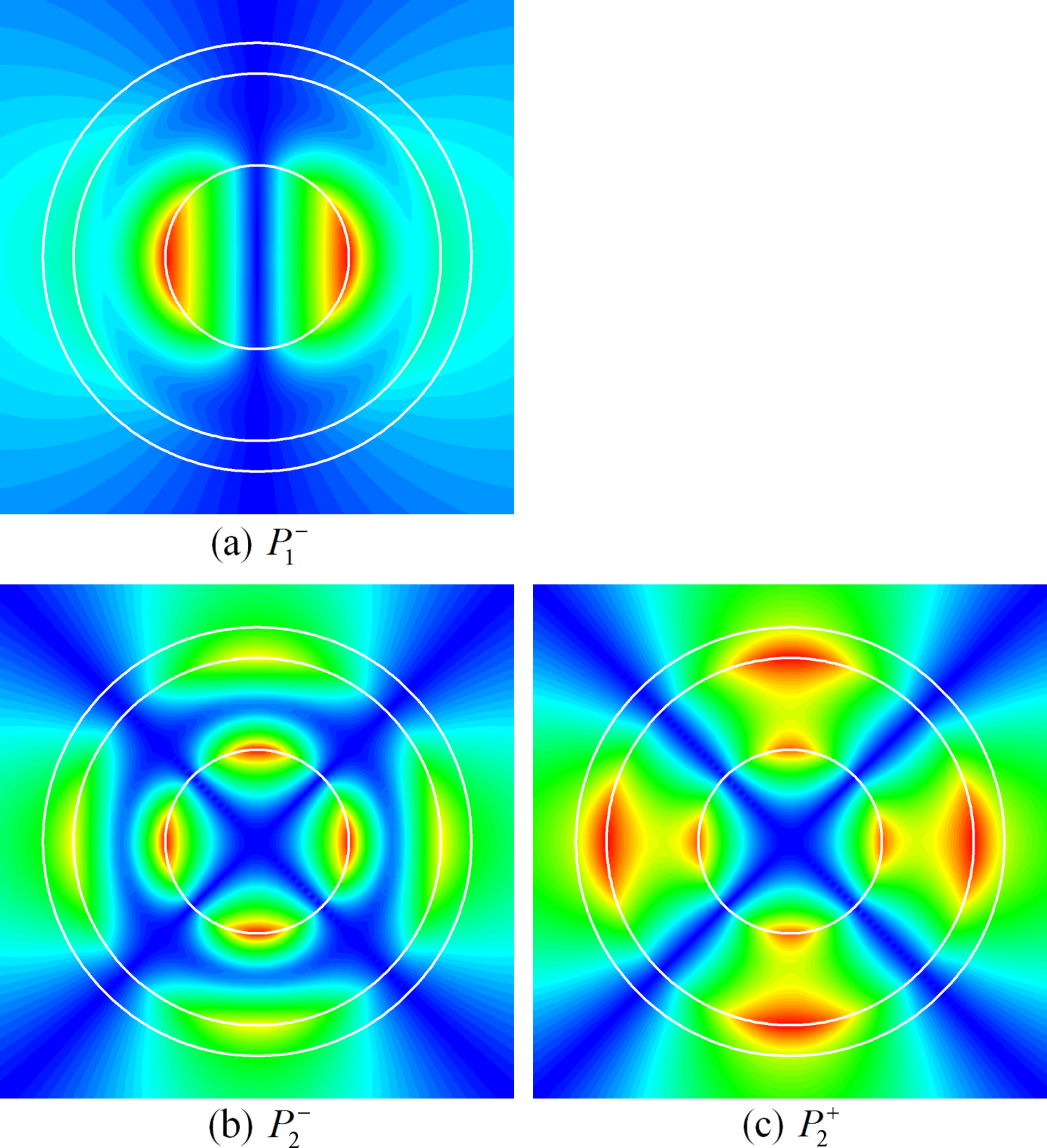
Near H-fields of the eigenmodes of thick-tube nanolaser, *a* = 30 nm, *d* = 10 nm, and *h* = 30 nm: (a) 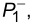 λ = 419.485 nm, γ = 0.137, (b) 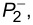 λ = 386.768 nm, γ = 0.1, (c) 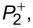 λ = 349.074 nm, γ = 0.283.

If the wall becomes thicker (i.e., *h* becomes larger), then 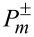 modes lose their hybrid features and transform to the LSP modes (of the same *m*) of the solid circular metal wire of radius *r* = *a* + *h* and of the void of radius *r* = *a* in the metal host medium, respectively. This is visible in the near-field portraits of the modes 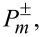 with azimuthal indices *m* = 1 and 2, of a thicker-wall nanotube laser, with *h* = 30 nm, shown in Figure 4.

Note that for both values of the tube wall thickness (10 nm in Figure 2 and 30 nm in Figure 4) the gain threshold value of the mode 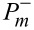 is lower than that of its sister mode 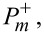 apparently because of the better overlap of its E-field with the active regions.

**Figure 5:**
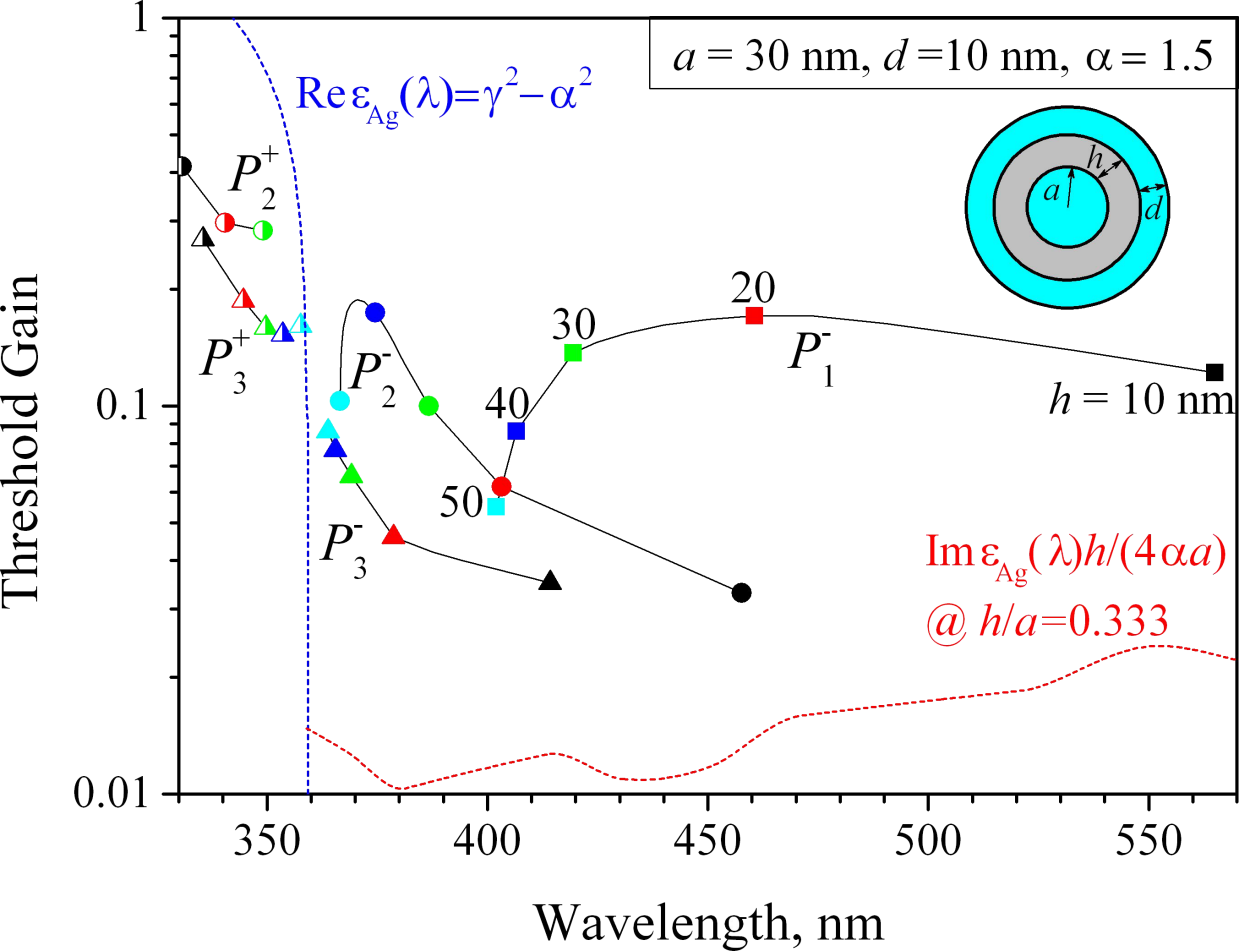
The trajectories of the eigenvalues of lasing modes on the plane (λ, γ) for plasmonic silver nanotube laser with an active core radius *a* = 30 nm and an active shell thickness *d* = 10 nm. The tube thickness varies from 10 to 50 nm as marked on the trajectories. The blue dashed line indicates the wavelength at which Re ε_met_(λ) = −α^2^ + γ^2^ (if γ = 0 then this is 359 nm [33]). The red dashed line indicates the lower-bound estimate (Equation 16) for the 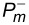 modes of silver tube with *h* = *a*/3.

To obtain a fuller vision of the dynamics of LEP eigenvalues on the plane (λ, γ) for the plasmonic silver nanotube laser, we have computed their trajectories for a device with an active core radius *a* = 30 nm and an active shell thickness *d* = 10 nm as the tube wall thickness *h* is varied from 10 nm to 50 nm (Figure 5).

We have studied only the modes that have their emission wavelengths in the visible range from 330 to 570 nm. Note that the trajectories have been calculated with small steps in the tube thickness to ensure their smoothness and the color markers are shown just for better understanding. The shape of the marker corresponds to the value of *m* and its color to the value of the varying tube thickness. For the 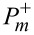 modes, the dots are half-filled, and for the 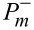 modes they are fully filled.

As can be seen from Figure 5, the “difference” mode 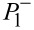 is the one with the highest red-shift, followed by the higher-order modes of the same type, 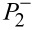 and 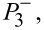 etc. If the tube gets thicker, each of the emission wavelengths of these modes moves closer to the value of 359 nm (from the red side) that is the accumulation point for the modes of the solid circular silver nanowire placed in the medium with α = 1.5. Note that, as already mentioned, if the tube thickness exceeds some 30 nm, then the nanotube modes lose their hybrid character and transfer to the modes of one of two boundaries: if *h*/*a* → ∞ then 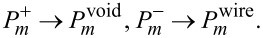 Indeed, the wavelengths of emission of the “sum” modes, 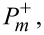 also come closer to 359 nm, albeit from the ultraviolet side.

The behavior of the thresholds as *h* is varied is more complicated. What is clearly visible from Figure 5 is that as far as *h* << *a*, the threshold of any hybrid mode 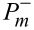 of the “difference” type is considerably lower than the corresponding threshold value for its sister mode of the “sum” type, 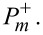 This is apparently explained by the better overlap of the mode E-field with the active region that becomes obvious for very thick tubes: the void modes fields are fully in the active core while the wire mode fields partially stretch out of the active shell.

Further, if the tube is thinner than the skin-depth (*h* < 20 nm), then for any fixed tube thickness the “difference” HLSP modes demonstrate lower threshold gains for the larger azimuth indices *m*. This is because their fields are more strongly confined near the outer surface of the silver tube. The thresholds of the “sum” modes, 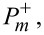 behave similarly. However, if the tube becomes thicker than the skin-depth, this rule no longer holds.

Note also that, under the variation of tube thickness *h*, the thresholds of the 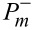 modes display a broad maximum, the position of which depends on *m*. This can be explained by the combined action of two competing mechanisms: in very thin tubes, the increase in thickness leads to the growth of ohmic losses in the silver as the E-fields of the 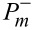 modes are non-zero functions of *r* inside the tube wall. However, gradually this growth of threshold is overcome by the better and better overlap of the almost purely void-mode E-field with the active core.

**Figure 6:**
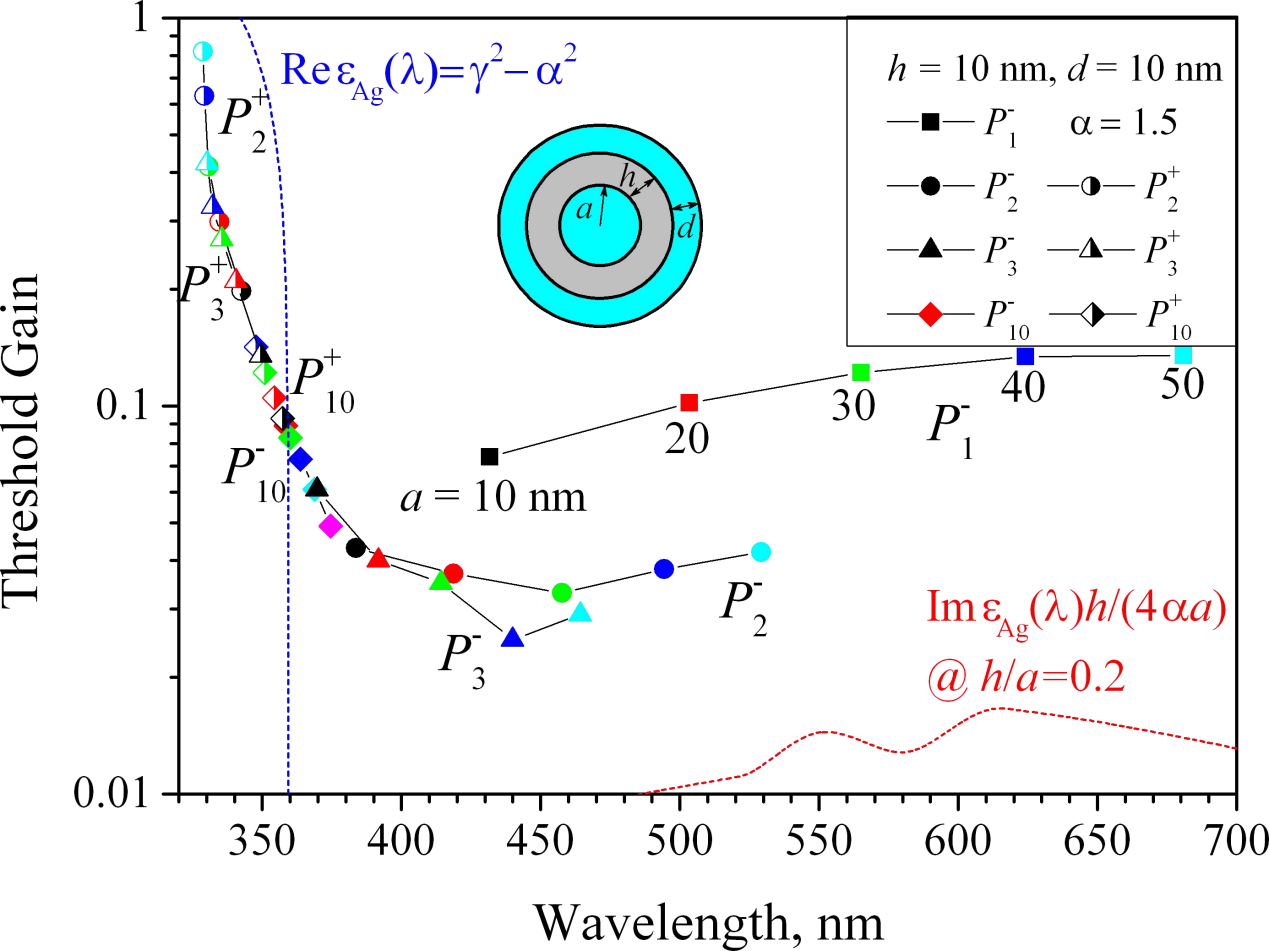
The trajectories of the eigenvalues of lasing modes on the plane (λ, γ) for a plasmon nanolaser with a silver tube thickness *h* = 10 nm and an active shell thickness *d* = 10 nm. The active core radius varies from 10 to 50 nm as marked on the trajectories. The dashed curves are the same as in Figure 5.

Figure 6 shows the trajectories of the eigenvalues of the HLSP modes 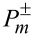 of the nanotube laser for *m* = 1, 2, 3, and 10 with the active-core radius *a* varying from 10 nm to 50 nm (with 10 nm step) and fixed *h* = *d* = 10 nm.

It is clearly seen that for the small-core nanolasers, the sister modes of the same azimuth index *m*, 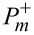 and 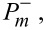 are close to each other, being shifted to the opposite sides of the curve ε_met_(λ) = −α^2^ + γ^2^. If the nanolaser core increases, their trajectories move in opposite directions, and for the 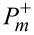 modes, the radiation wavelengths of which are in the region of higher losses in silver, the threshold significantly increases, reaching 0.8–0.9 at *a* = 50 nm. The threshold values of the 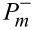 modes demonstrate better stability, and have much lower values than those of the 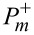 sister modes. The minimum value of γ is demonstrated by the hexapole “difference” mode 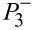 for the nanotube laser with *a* = 30 nm (blue triangle in Figure 6): γ = 0.025 at λ = 439.89 nm.

**Figure 7:**
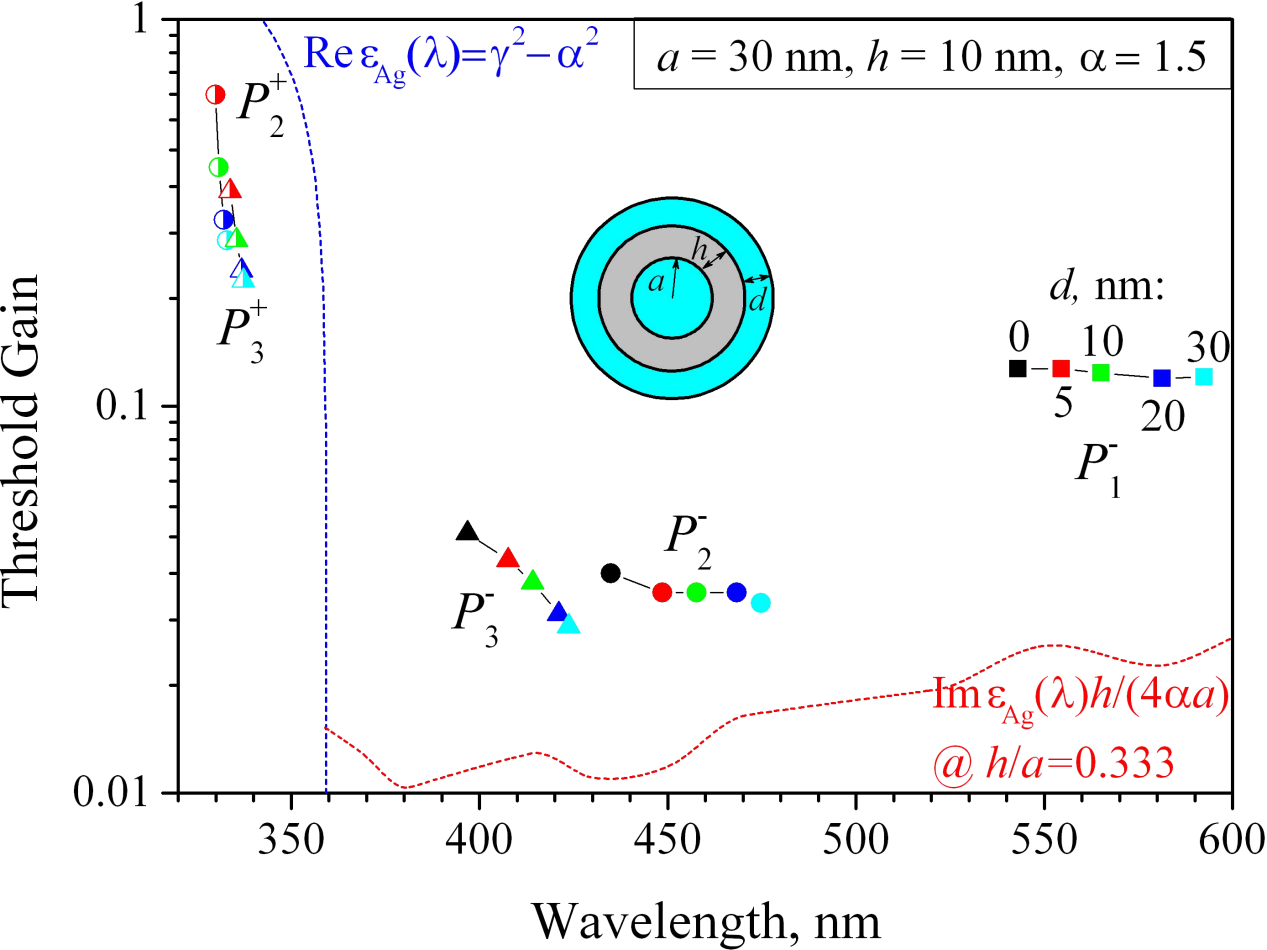
The trajectories of the eigenvalues of lasing modes on the plane (λ, γ) for a plasmonic silver nanotube laser with active core radius *a* = 30 nm and tube thickness *h* = 10 nm. The active shell thickness varies from 0 to 30 nm as marked on the trajectories. The dashed curves are the same as in Figure 5 and Figure 6.

Finally, Figure 7 shows the trajectories of the eigenvalues of HLSP modes 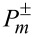 of the nanotube laser for *m* = 1, 2 and 3, with fixed active core radius *a* = 30 nm and tube thickness *h* = 10 nm but with the active-shell thickness *d* varying from 0 to 30 nm. Note that there is no “sum” dipole mode 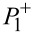 in the range of wavelengths studied, as it is shifted to the far ultraviolet and has very high threshold gain.

As can be seen in Figure 7, an increase of the shell thickness leads to red-shifts of all mode wavelengths because the active material is optically denser than free space. Furthermore, all modes demonstrate a decrement of their thresholds that is more pronounced for the “sum” modes 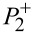 and 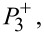 and the higher-order “difference” mode 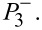 This is obviously explained by the better overlap of the mode E-fields with the thicker active shell. A further increase of the shell thickness does not lead to a significant change in the thresholds, but brings the shell modes into the visible range of wavelengths when *d* approaches λ/(2α) – that is around 100 nm in the case studied here.

## Conclusion

We have presented the results of the classical electromagnetics analysis of the LEP eigenvalues, i.e., the emission wavelengths and the threshold gains, of the hybrid plasmon modes of a silver nanotube laser. To the best of our knowledge, such analysis has not been done before.

Summarizing, we can say that the optimal nanotube laser configuration looks like a 30 nm to 50 nm active core of a 10 nm thick silver tube, covered with a 10 nm active shell. This is because making the core larger than 50 nm in radius shifts the emission of the working mode to the infrared and making it larger than 100 nm in radius brings the first core mode to the visible range. Making the nanotube wall thicker than 20 nm spoils the hybridization of the LSP modes and pushes all of them together, at blue and violet wavelengths, while selecting it much thinner than 10 nm may lead to the increment of losses due to non-local effects. Using the active shell thicker than 10 nm has little effect on the thresholds of the working modes, while making it close to 100 nm brings the unwanted first shell mode to the visible part of the spectrum.

The presented studies reveal that the dipole difference-type HLSP mode 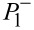 is the most attractive for being selected as the working mode. This is because it has a relatively low threshold gain (around γ = 0.1 if *h* = 10 nm) and emits light in the yellow or green parts of the visible spectrum. It is well separated from the higher-order modes of the same type, 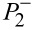 and 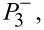 which are significantly shifted to the blue range. This seems to be more important than the fact that the latter two modes have 2–3 times lower values of threshold gain index than 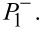 All modes of the sum type, 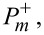 emit light in the ultra-violet part of the spectrum and have roughly 10 times higher thresholds than their difference-type sisters. Therefore they are apparently not interesting for applications.

We believe that the new results presented will help in the design of nanotube lasers, which are essentially single-mode sources.
